# Structural and Electronic Properties of Hexagonal and Cubic Phase AlGaInN Alloys Investigated Using First Principles Calculations

**DOI:** 10.1038/s41598-019-43113-w

**Published:** 2019-04-29

**Authors:** Y.-C. Tsai, C. Bayram

**Affiliations:** 1 Department of Electrical and Computer Engineering, University of Illinois at Urbana, Champaign, Illinois 61801 USA; 2 Micro and Nanotechnology Laboratory, University of Illinois at Urbana, Champaign, Illinois 61801 USA

**Keywords:** Electronic properties and materials, Electrical and electronic engineering, Electronic properties and materials, Computational science

## Abstract

Structural and electronic properties of hexagonal (h-) and cubic (c-) phase AlGaInN quaternary alloys are investigated using a unified and accurate local-density approximation-1/2 approach under the density-functional theory framework. Lattice bowing parameters of h- (and c-) phase AlGaN, AlInN, InGaN, and AlGaInN alloys are extracted as 0.006 (−0.007), 0.040 (−0.015), 0.014 (−0.011), and −0.082 (0.184) Å, respectively. Bandgap bowing parameters of h- (and c-) phase AlGaN, AlInN, InGaN, and AlGaInN alloys are extracted as 1.775 (0.391), 3.678 (1.464), 1.348 (1.164), and 1.236 (2.406) eV, respectively. Direct-to-indirect bandgap crossover Al mole fractions for c-phase AlGaN and AlInN alloys are determined to be 0.700 and 0.922, respectively. Under virtual crystal approximation, electron effective masses of h- and c-phase AlGaInN alloys are extracted and those of c-phase alloys are observed to be smaller than those of the h-phase alloys. Overall, c-phase AlGaInN alloys are shown to have fundamental material advantages over the h-phase alloys such as smaller bandgaps and smaller effective masses, which motivate their applications in light emitting- and laser diodes.

## Introduction

Ever since the inventions of high-efficiency and high-brightness blue and white light emitting diodes (LEDs), hexagonal (h-) phase (wurtzite) gallium nitride (GaN) and its ternary alloys–AlGaN, AlInN, and InGaN–have successfully revolutionized the way we generate efficient light source, which spans all across visible light and extends in ultraviolet spectra^1^. To date, the feasible way to generate a variety of visible light, including natural white light, is to superimpose the primary colors of light: red (1.91 eV), green (2.24 eV), and blue (2.75 eV), so-called full-color tuning. Highly-efficient red and blue LEDs are fabricated by AlGaInP and InGaN materials, respectively; while, green LEDs are manufactured by phosphor-coated blue LED, phosphide-based materials, or nitride-based materials^2^. However, the efficiency of green LEDs is still more than twice less efficient than that of the red and blue LEDs due to the energy loss of phosphor-coated blue LED during wavelength conversion and the indirect bandgap of phosphide-based materials. The factors that plagued nitride-based green LEDs are more complicated; to be simplified, the strong spontaneous polarization and piezoelectric polarization of h-phase nitrides resulting from the lack of inversion symmetry and the lattice mismatch under high-In alloying deteriorate the radiative recombination efficiency, the phenomenon is known as quantum-confined Stark effect^3^. As a result of lacking suitable materials for green emitters, the low efficiency of green LEDs is described as a “green gap”. An urgent engineering challenge is to discover a material that has a compatible and direct bandgap to emit green wavelength, meanwhile, depress the quantum-confined Stark effect.

III-nitrides can also crystallize in the cubic (c-) phase (zincblende) structure, which has potential advantages in optical and electrical applications. Specifically, c-phase GaN exhibits high electron mobility^4^, smaller acceptor activation energy^5^, high hole mobility^6^, and small Auger losses^7^. Additionally, the c-phase III-nitrides are centrosymmetric, which makes them polarization-free in the growth <001> direction. This leads to a larger electron-hole wavefunction overlap, which increases radiative recombination efficiency and optical gain^8^. Thus, c-phase GaN and its ternary and quaternary alloys are ideal photonic materials for high-efficiency vertical transport devices, which includes light emitting- and laser diodes. However, due to the metastability of c-phase III-nitrides, the crystal structure suffers from a high level of defectivity and can easily relax to the h-phase structure. Recently, Liu *et al*. have synthesized c-phase GaN with high crystal-quality and high phase-purity using the U-groove hexagonal-to-cubic phase transition approach. A band-to-band emission in the ultraviolet (UV) from the c-phase GaN has shown a record high internal quantum efficiency of 29%, which is higher than that of bulk h-phase GaN at 12%^9^. Further increase of the radiative efficiency using carrier confinement necessitates the use of quantum well structures. Yet, there are limited computational and experimental studies on the structural and electronic properties of these c-phase III-nitrides; there are no numerical expressions for the bandgap, lattice-constant, and effective mass of c-phase III-nitrides as well. In literature, bandgaps, direct-to-indirect bandgap crossover points, and effective masses of h- and c-phase AlN, GaN, and InN are studied using various simulation approaches such as local-density approximation (LDA)^10,11^, G_0_W_0_ approximation^12^, and generalized gradient approximation (GGA) approaches^13^, which led to dissimilar bandgap values. For instance, the calculated bandgap of h-phase InN ranges from 0.69^12^ and 1.02^13^ to 2.00^11^ eV; the broad range of bandgap is also reported in experiments, while the widely accepted value is 0.78 eV^14^. The inconsistent bandgap can virtually affect the accuracy in determining the bandgap of In-rich ternary and quaternary III-nitrides, which alters the bowing parameters and the direct-to-indirect bandgap crossover points^15^. In addition, the evolution of electron effective mass in c-phase ternary and quaternary III-nitrides with respect to Al, Ga, and In mole fractions is still unclear.

**Figure 1:**
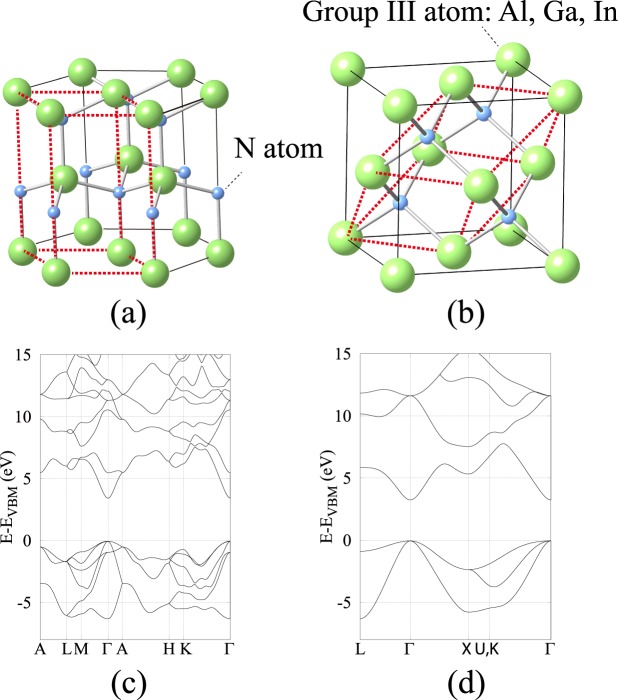
Illustrations of a unit cell of (**a**) h- and (**b**) c-phase binary III-nitrides. Black solid lines outline the contour of unit cells; while, the red dash lines highlight the corresponding primitive cells. The primitive cell of h-phase binary III-nitrides contains 2 group III atoms and 2 N atoms; while, the primitive cell of c-phase binary III-nitrides contains 1 group III atom and 1 N atom. Demonstrations of the electronic structure of (**c**) h- and (**d**) c-phase GaN along the high-symmetry lines calculated by LDA-1/2 method. E_VBM_ is the energy of valence band maximum.

In this work, lattice constants and bandgaps of h- and c-phase III-nitrides are investigated under the density-functional theory framework. For the crystal relaxation, LDA is applied to approximate the exchange-correlation energy of the many-electron system due to its reliability and low computational cost of determining the ground-state electronic properties. However, it is well-known that LDA underestimates the bandgap since the exchange-correlation potential is not discontinuous between the conduction and valence band. LDA-1/2 approach, on the other hand, is applied to correct the bandgap by half-ionizing electron to conduction band with the same reliability and computational cost as the LDA method. It can retrieve the accurate bandgap of III-V alloys because the relation between the single-particle energy obtained from Kohn-Sham equation and the electron occupation can be linearly approximated^16^. Figure 1(a) illustrates a unit cell of h-phase binary III-nitrides. The red dash lines highlight the primitive cell that contains 2 group III atoms and 2 N atoms and defines the h-phase crystal structure. Similarly, Fig. 1(b) shows a unit cell of c-phase binary III-nitrides, where the primitive cell contains 1 group III atom and 1 N atom. Figure 1(c,d) demonstrate the electronic structure of h- and c-phase GaN along the high-symmetry lines, where the energy states shift with respect to the valence band maximum (E_VBM_). The electronic structure of ternary (AlGaN, AlInN, and InGaN) and quaternary (AlGaInN) alloys with different mole fractions are calculated by substituting the group III atoms in a unit cell, which allows an accurate interpolation of direct-to-indirect bandgap crossover points, an extraction of bowing parameters, and an numerical expression of lattice constants and bandgaps. Transverse and longitudinal electron effective masses are also extracted for h- and c-phase III-nitrides.

## Methods

**Figure 2:**
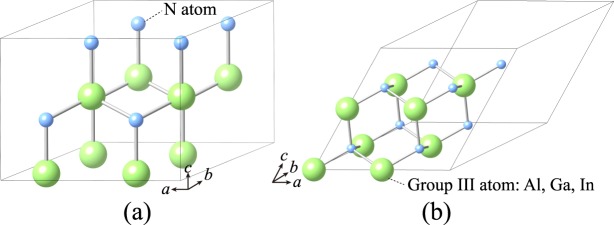
Illustrations of (**a**) 2 × 2 × 1 and (**b**) 2 × 2 × 2 primitive cells of h- and c-phase binary III-nitrides used to construct and simulate 16-atom supercells of h- and c-phase quaternary alloys, respectively.

First-principles calculations are carried out under the density-functional theory framework implemented in the Vienna *ab initio* Simulation Package (VASP)^17^ and are carried out on binary, ternary and quaternary III-nitrides. Al 3*s*^2^3*p*^1^, Ga 4*s*^2^4*p*^1^, In 4*d*^10^5*s*^2^5*p*^1^, and N 2*s*^2^2*p*^3^ valence electrons are characterized using projector augmented wave pseudopotentials (PAW)^18^; while the cut-off kinetic energy of 500 eV is secured for the plane-wave expansion. All atoms are fully relaxed so that the interatomic forces and energy difference are smaller than 0.01 eVÅ^−1^ per ion and 10^−6^ eV/atom, respectively. Figure 2(a) illustrates 2 × 2 × 1 primitive cell of h-phase binary III-nitrides used to construct and simulate a 16-atom supercell of h-phase quaternary alloys. Similarly, Fig. 2(b) illustrates 2 × 2 × 2 primitive cell of c-phase binary III-nitrides used to construct and simulate a 16-atom supercell of c-phase quaternary alloys. Each supercell contains 8 group III atoms and 8 N atoms. The mole fractions of Al, Ga, and In can be adjusted by substituting the 8 group III atoms in the supercell, which gives the finest mole fraction tunability of 0.125. For instance, the unit cell of Al_0.375_Ga_0.625_N contains 3 Al atoms, 5 Ga atoms, and 8 N atoms; while, the unit cell of Al_0.5_Ga_0.5_N has 4 Al atoms, 4 Ga atoms, and 8 N atoms. Overall, 9 and 45 cases are sampled individually for each phase of ternary (AlGaN, AlInN, and InGaN) and quaternary (AlGaInN) alloys. Although the initial size of supercells used to build ternary and quaternary alloys is identical, the size of supercells changes after the atoms and the cells are relaxed to its equilibrium positions and volumes. For instance, h- and c-phase AlN have the smallest supercells of 163.36 and 163.75 Å^3^; while h- and c-phase InN have the largest supercells of 240.58 and 240.93 Å^3^, respectively. The Brillouin zone of the supercells is sampled with an 8 × 8 × 8 gamma-centered Monkhorst-Pack set of *k*-points. Notably, the bandgap of binary alloys (AlN, GaN, and InN) calculated by the supercell approach is consistent with the bandgap calculated using the primitive cells, which indicates the numerical accuracy of the supercell calculations. LDA is performed to calculate the exchange-correlation energy of many-electron system for structural relaxation. To fix the bandgap underestimation of LDA, LDA-1/2 method is utilized to calculate the electronic structure and bandgap of III-nitrides. LDA-1/2 method inherits from Slater half-occupation scheme^16,19^, which assumes that single-particle Kohn-Sham energy linearly depends on the occupation expressed as:

$$E(N)-E(N-1)={\int }_{N-1}^{N}\frac{\partial E(x)}{\partial x}dx\approx {\varepsilon }_{\alpha }(N-0.5),$$

where *E*(*N*) and *E*(*N*−1) are the total system energies under the occupation number of *N* and *N*−1, respectively; *ε*_*α*_ is the single-particle Kohn-Sham energy at state α. Using the linear dependence of the single-particle Kohn-Sham energy on the occupation number, the ionization energy is approximated by the single-particle Kohn-Sham energy with a half ionization, as shown in the last term. Starting from this postulation, Ferreira *et al*. further demonstrated that the single-particle Kohn-Sham energy with a half ionization is equivalent to the single-particle Kohn-Sham energy at ground-state, *ε*_*α*_(*N*), minus a hole self-energy (*S*_*α*_) at state α expressed as^20^:

$${\varepsilon }_{\alpha }(N-0.5)={\varepsilon }_{\alpha }(N)-{S}_{\alpha }$$

*S*_*α*_ can be formulated by quantum-mechanical average:

$${S}_{\alpha }=\int d\overrightarrow{r}\cdot {n}_{\alpha }(\overrightarrow{r}){V}_{s}(\overrightarrow{r}){\rm{\Theta }}(|\overrightarrow{r}|),$$

where *n*_*α*_, *V*_*s*_, and Θ are the electron density at state α, the self-energy potential, and the trim function, respectively. The trim function is used to cut the Coulomb tail of *V*_*s*_ in an infinite crystal, which has a negligible contribution to the self-energy due to the localization of wavefunction, defined by:

$${\rm{\Theta }}(|\overrightarrow{r}|)=\{\begin{array}{ll}A{[1-{(\frac{|\overrightarrow{r}|}{CUT})}^{n}]}^{3} & |\overrightarrow{r}|\le CUT\\ 0 & |\overrightarrow{r}| > CUT\end{array}$$

where *CUT* and *n* are the cutoff radius and cutoff sharpness; while, *A* is the amplitude of trim function, which can be exploited to adjust the amplitude of self-energy and bandgap linearly and semi-empirically. The bandgap (*E*_*g*_), defined by the energy difference between the system energy that has one electron excited from the valence to the conduction band, *E*(*N*−1, 1), and the ground-state system energy, *E*(*N*,0), can be formulated as the bandgap calculated by LDA (${E}_{g}^{LDA}$) with corrections from electron (*S*_*c*_) and hole (*S*_*v*_) self-energies:

$${E}_{g}=E(N-1,1)-E(N,0)=[{\varepsilon }_{c}(N)-{S}_{c}]-[{\varepsilon }_{v}(N)-{S}_{v}]={E}_{g}^{LDA}+{S}_{v}-{S}_{c},$$

where *ɛ*_*c*_ and *ɛ*_*v*_ are single-particle Kohn-Sham energies for electron and hole, respectively.

According to the suggestions by Ferreira *et al*., *A* = 1 and *n* = 8 are fixed in the first place^20^. The *CUT* values of 4.40, 1.53, and 3.0Å are benchmarked for Al, Ga, and N ions to make the bandgap of h-phase AlN (6.2 eV) and h-phase GaN (3.5 eV) experimentally-verified^14^. However, several combinations of *CUT* and *n* have been benchmarked for In ion, none of them gives a satisfactory result. The optimal bandgap is 1.24 eV, which is similar to other reports^13,15,21^. But, it significantly deviates from the experiment of 0.78 eV. The bandgap overestimation may originate from the assumption that the ionization energy is equal to the single-particle Kohn-Sham energy with a half ionization. In other words, the single-particle Kohn-Sham energy of In ion may not linearly dependent upon the occupation number. To determine the correct self-energy, the trim function amplitude *A* is benchmarked semi-empirically to adjust the self-energy amplitude and the bandgap linearly. Finally, *A* = 2.3 and *CUT* = 2.926Å are benchmarked for In ion.

To model the electronic properties of multinary alloys, the unit cell of multinary alloys is constructed by the supercell method. However, the corresponding Brillouin zone is folded with the increasing size of the supercell. As the consequence, it is challenging to analyze the electronic properties, such as extracting effective mass and determining indirect bandgap, from the *E-k* dispersion because the energy states outside of the first Brillouin zone are folded into the first Brillouin zone so-called band folding. *fold2Bloch* utility^22^ is employed to unfold the band structure of supercell back to its primitive basis representation by calculating and filtering the Bloch spectral density, the procedure is known as virtual crystal approximation. After unfolding the electronic structure, the curvatures of the lowest conduction band for electron effective mass calculations are extracted using the finite-difference method and parabolic approximation. The lattice constants, bandgaps, transverse (${m}_{t}^{\ast }$) and longitudinal (${m}_{l}^{\ast }$) electron effective masses for h- and c-phase III-nitrides with the corresponding corroboration from experiments are tabulated (Table 1). For h-phase alloys, ${m}_{t}^{\ast }$ and ${m}_{l}^{\ast }$ are calculated along [100] and [001] directions. For c-phase alloys, ${m}_{l}^{\ast }$ is computed along [101] direction and ${m}_{t}^{\ast }$ is calculated along [111] direction, at Γ-valley (direct bandgap alloys), or [211] direction, at X-valley (for indirect bandgap alloys).

**Table 1:** Lattice constants along [100] (*a*) and [001] (*c*) directions, bandgaps, and electron effective masses of h- and c-phase binary III-nitride alloys are tabulated with published experimental data, extracted from ref.^14^. unless noted otherwise.

Phase		*a*(Å)	*a*_*exp*_(Å)	*c*(Å)	*c*_*exp*_(Å)	*E*_*g*_(eV)	${{\boldsymbol{E}}}_{{\boldsymbol{g}},\exp }({\bf{e}}{\bf{V}})$	${{\boldsymbol{m}}}_{{\boldsymbol{t}}}^{\ast }({{\boldsymbol{m}}}_{0})$	${{\boldsymbol{m}}}_{{\boldsymbol{t}},\exp }^{\ast }\,({{\boldsymbol{m}}}_{0})$	${{\boldsymbol{m}}}_{{\boldsymbol{l}}}^{\ast }\,({{\boldsymbol{m}}}_{0})$	${{\boldsymbol{m}}}_{l{\boldsymbol{,}}\exp }^{\ast }\,({{\boldsymbol{m}}}_{0})$
Hexagonal	AlN	3.09	3.11	4.94	4.98	6.30	6.25	0.327	0.29‒0.45^35^	0.314	0.29‒0.45^35^
	GaN	3.18	3.19	5.18	5.19	3.47	3.51	0.193	0.20	0.172	0.20
	InN	3.50	3.55	5.66	5.70	0.77	0.78	0.038	0.039^36^	0.046	0.047^36^
Cubic	AlN	4.34	4.38	—	—	5.26	5.30^37^	0.327^a^	0.31^a,b^	0.529^a^	0.53^a,b^
	GaN	4.50	4.50	—	—	3.27	3.30	0.175	0.15	0.175	0.15
	InN	4.94	4.98	—	—	0.62	0.60^38^	0.064	0.07	0.064	0.07

^a^At X-valley.

^b^Average value from multiple theoretical sources.

## Results and Discussion

**Figure 3:**
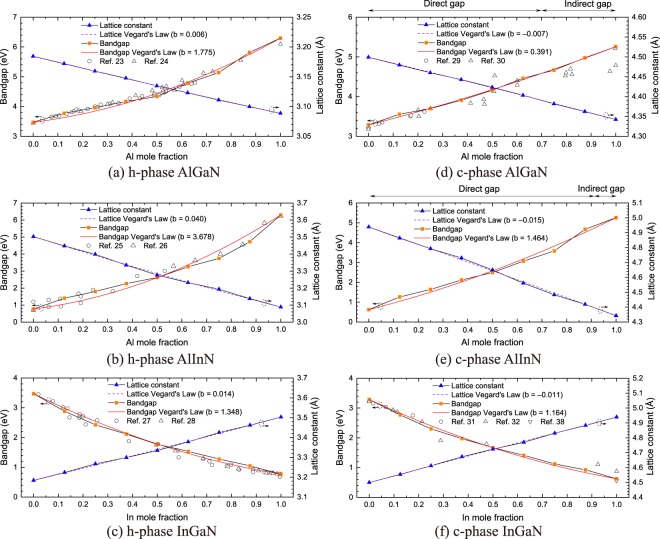
Lattice constants, lattice bowing parameters, bandgaps, and bandgap bowing parameters of h-phase (**a**) AlGaN, (**b**) AlInN, (**c**) InGaN, and c-phase (**d**) AlGaN, (**e**) AlInN, and (**f**) InGaN alloys. Direct-to-indirect bandgap Al mole fraction crossover points in c-phase AlGaN and AlInN alloys are calculated as 0.700 and 0.922, corresponding to the bandgap energies of 4.585 and 4.792 eV, respectively.

Figure 3(a–f) summarizes the lattice constants along [100] direction, bandgaps, and fits for both lattice constant and bandgap using Vegard’s law of h- [Fig. 3(a–c)] and c-phase [Fig. 3(d–f)] AlGaN, AlInN, and InGaN. All six ternary alloys have a linear relationship between lattice constants and Al or In mole fractions. The lattice bowing parameters of h- (and c-) phase AlGaN, AlInN, and InGaN alloys are extracted as 0.006 (−0.007), 0.040 (−0.015), and 0.014 (−0.011) Å fitted by lattice Vegard’s law tabulated in Table 2. Since the defectivity and piezoelectric polarization of III-nitrides strongly depend on the lattice mismatch between two substrates, it is essential to estimate the lattice mismatch between the potential materials of quantum well and GaN. The lattice mismatch can be estimated by $\frac{a-{a}_{GaN}}{{a}_{GaN}}\times 100 \% ,$ where *a* and *a*_*GaN*_ are the lattice constants of the potential material and GaN, respectively. As the results, the strains of h-phase AlN, c-phase AlN, h-phase InN, and c-phase InN interfaced with GaN are −3.00%, −3.49%, 10.00%, and 9.77%, respectively. The strains between the ternary alloys and GaN can be linearly interpolated due to the linear relationship between lattice constants and Al and In mole fractions.

**Table 2:** Lattice and bandgap bowing parameters of h- and c-phase ternary III-nitrides.

Property	Phase	Alloy	*b*
Lattice constant	Hexagonal	AlGaN	0.006
		AlInN	0.040
		InGaN	0.014
	Cubic	AlGaN	−0.007
		AlInN	−0.015
		InGaN	−0.011
Bandgap	Hexagonal	AlGaN	1.775
		AlInN	3.678
		InGaN	1.348
	Cubic	AlGaN	0.391
		AlInN	1.464
		InGaN	1.164

The generic Vegard’s law for ternary alloys is expressed as: ${P}_{{A}_{x}{B}_{1-x}N}=x{P}_{AN}+(1-x){P}_{BN}-bx(1-x)$, where ${P}_{{A}_{x}{B}_{1-x}N}$, *P*_*AN*_, and *P*_*BN*_ are the property (*P*) of ternary and binary nitrides, respectively, and *b* is the bowing parameter.

The accuracy of LDA-1/2 method on the bandgap calculations of ternary alloys is demonstrated by comparing the simulation results with the experimental measurements^23–32^, except for c-phase AlInN and In-rich c-phase InGaN due to the lack of experimental reference data. The nonlinear dependence of bandgap on the Al or In mole fraction is summarized using bandgap Vegard’s law tabulated in Table 2. The bandgap bowing parameters of h-phase AlGaN, AlInN, and InGaN are extracted as 1.775, 3.678, and 1.348 eV, respectively, which are larger than their c-phase counterparts of 0.391 (AlGaN), 1.464 (AlInN), and 1.164 eV (InGaN). The simulated bandgaps agree well with the available experiments. It is worth mentioning that most of the exchange-correlation functionals, including highly-accurate hybrid functionals and GW quasi-particle approximation, fail to recover the bandgap of InN^21^, which also leads to the bandgap overestimation of In-rich h-phase InGaN by more than 20%^15^. The similar issue also occurs in the LDA-1/2 method. To address this issue, the self-energy energy of In ion is linearly and semi-empirically increased by 2.3 times so that the bandgap of h-phase InN matches the experiment of 0.78 eV. The numerical adjustment corrects the underestimation of self-energy and, eventually, improves the bandgap overestimation. Therefore, the correction of self-energy using h-phase InN bandgap fixes the bandgap overestimation of In-rich h-phase InGaN. The calculated bandgap of c-phase InN also matches the experiment, which further indicates the consistency and reliability of the semi-empirical correction.

The electronic structures of c-phase AlGaN and c-phase AlInN are unfolded to examine the crossover point at which the direct-to-indirect bandgap transition occurs. The evolutions of conduction band minimum and valence band maximum are traced with respect to Al mole fraction. It is observed that the conduction band minimum at X-valley decreases as the Al mole fraction increases; while, the conduction band minimum at Γ-valley shifts oppositely. The crossover points are interpolated using the energy difference of conduction band minimum at the X-valley and the Γ-valley. When the Al mole fraction reaches the crossover point of 0.700 and 0.922 for c-phase AlGaN and c-phase AlInN, respectively, the X-valley takes the possession of conduction band minimum, which indicates indirect bandgap. The crossover point of c-phase AlGaN agrees well with the reported value of 0.692^33^. However, the crossover point of c-phase AlInN is slightly greater than the reported values of 0.81‒0.85^34^, which are calculated based on the overestimated bandgap of c-phase InN. However, because the LDA-1/2 approach fully recovers the deeply-bend Γ-valley contributed from c-phase InN, a higher content of c-phase AlN is required to lift up the Γ-valley and drag down the X-valley. Ultimately, the c-phase AlGaN and AlInN turn into indirect-gap alloys as the Al mole fraction goes beyond 0.700 and 0.922, which correspond to the bandgap energies of 4.585 and 4.792 eV, respectively.

**Figure 4:**
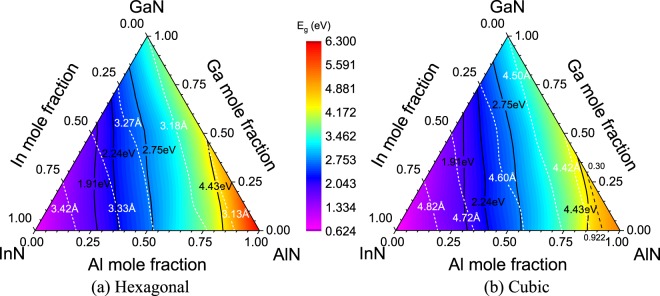
Lattice constants (white dashed lines) and bandgaps of (**a**) h-phase and (**b**) c-phase AlGaInN alloys are plotted. The bandgap boundary (black dashed line) between the direct and indirect c-phase Al_*x*_Ga_*y*_In_*1−x−y*_N alloys obey *y* = –1.351*x* + 1.246, where *x* and *y* are the Al and Ga mole fractions. Black solid lines highlight the bandgaps of common emitters: red (1.91 eV), green (2.24 eV), blue (2.75 eV), and UV (4.43 eV).

Figure 4(a,b) plot the lattice constants (white dashed lines) and bandgaps (color contour and black solid lines) of h- and c-phase Al_*x*_Ga_*y*_In_*1−x−y*_N, respectively. Lattice Vegard’s law for quaternary alloys is used to have a numerical expression for the lattice constants of quaternary alloys. The lattice bowing parameters of h- (and c-) phase AlGaInN are extracted to be −0.082 (0.184) Å with R^2^ values of 0.998 (0.998). The lattice constants of h- and c-phase Al_*x*_Ga_*y*_In_*1−x−y*_N are summarized in the unit of Å by the numerical expressions tabulated in Table 3. It worth mentioning that the h- (and c-) phase quaternary alloys obeying *y* = −1.325*x* + 1 (*y* = −1.350*x* + 1) are lattice matched to GaN.

**Table 3:** Lattice and bandgap bowing parameters of h- and c-phase AlGaInN and effective mass bowing parameter of h-phase Al_*x*_Ga_*y*_In_*1−x−y*_N.

Property	Phase	*B*	Numerical expression
Lattice constant	Hexagonal	−0.082	$0.040{x}^{2}-0.082{x}^{2}y-0.082x{y}^{2}+0.131xy+0.014{y}^{2}-0.454x-0.332y+3.503$
	Cubic	0.184	$-0.015{x}^{2}+0.184{x}^{2}y+0.184x{y}^{2}-0.203xy-0.011{y}^{2}-0.582x-0.429y+4.939$
Bandgap	Hexagonal	1.236	$3.678{x}^{2}+1.236{x}^{2}y+1.236x{y}^{2}+2.015xy+1.348{y}^{2}+1.853x+1.355y+0.769$
	Cubic	2.406	$1.464{x}^{2}+2.406{x}^{2}y+2.406x{y}^{2}-0.170xy+1.164{y}^{2}+3.171x+1.498y+0.624$
Effective mass	Hexagonal	−0.4684	$0.0217{x}^{2}-0.4684{x}^{2}y-0.4684x{y}^{2}+0.6159xy+0.1252{y}^{2}+0.2672x+0.0296y+0.0382$

The generic Vegard’s law for quaternary alloys is expressed as: $$P(x,y)=x{P}_{{AlN}}+y{P}_{{GaN}}+(1-x-y){P}_{{InN}}-{b}_{{AlInN}}x(1-x)-{b}_{{InGaN}}y(1-y)-{b}_{{AlGaN}}xy+({b}_{{InGaN}}+$$$${b}_{{AlInN}})xy-Bxy(1-x-y)$$ $$,$$ where *P*(*x, y*), *P*_*AIN*_, *P*_*GaN*_, and *P*_*InN*_ are the property (*P*) of Al_*x*_Ga_*y*_In_*1−x−y*_N, AlN, GaN, and InN; while, $${b}_{AlGaN}$$, $${b}_{AlInN}$$, $${b}_{InGaN}$$, and *B* are the corresponding bowing parameters of AlGaN, AlInN, InGaN, and AlGaInN, respectively.

The bandgaps of h-phase quaternary alloys are 19.77% (AlN) and 22.58% (InN) larger than their c-phase counterparts. Similarly, bandgap Vegard’s law is applied for the numerical expression of quaternary alloy bandgap. The bandgap bowing parameters of h- and c-phase AlGaInN are extracted to be 1.236 and 2.406 eV with R^2^ values of 0.968 and 0.989, respectively, where the numerical expressions of the bandgaps in the unit of eV are tabulated in Table 3 for h- and c-phase AlGaInN. Notably, the bandgap boundary (black dashed line) between the direct and indirect c-phase Al_*x*_Ga_*y*_In_*1−x−y*_N alloys obey *y* = −1.351*x* + 1.246, where *x* and *y* are the Al and Ga mole fractions defined within [0, 1]. Black solid lines highlight the bandgaps of common emitters: red (1.91 eV), green (2.24 eV), blue (2.75 eV), and UV (4.43 eV). For engineering applications, the h- (and c-) phase quaternary alloys obeying *y* = −1.829*x* + 0.678 (*y* = −1.752*x* + 0.726) have the bandgap of 2.24 eV that is promising for green emitters; while, *y* = −1.587*x* + 0.838 (*y* = −1.555*x* + 0.867) guarantees the bandgap of 2.75 eV for blue emitters. For UV emitters, h- (and c-) phase quaternary alloys obeying *y* = −1.550*x* + 1.297 (*y* = −2.407*x*^2^ + 1.947*x* + 0.095) satisfies the bandgap of 4.43 eV, where *x* is defined within [0, 1] ([0.624, 0.855]). The mole fractions for green, blue, and UV emitters can be further optimized by minimizing the lattice mismatch. As the results, h- (and c-) phase In_0.322_Ga_0.678_N (In_0.274_Ga_0.726_N), In_0.162_Ga_0.838_N (In_0.133_Ga_0.867_N), and Al_0.837_In_0.163_N (Al_0.855_In_0.145_N) are the best candidates for green, blue, and UV emitters in terms of the matched bandgap and have the least lattice-mismatch with GaN, respectively.

**Figure 5:**
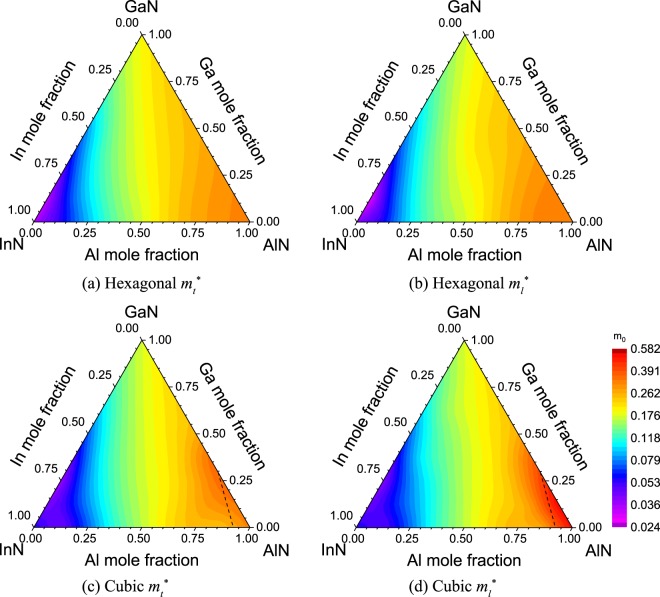
(**a**,**c**) ${m}_{t}^{\ast }$ and (**b**,**d**) ${m}_{l}^{\ast }$ of h- and c-phase AlGaInN alloys are plotted, respectively. The black dashed line obeying *y* = −1.351*x* + 1.246 marks the boundary between the direct and indirect c-phase Al_*x*_Ga_*y*_In_*1−x−y*_N alloys.

Figure 5(a–d) plots the anisotropic electron effective masses of h- and c-phase AlGaInN. ${m}_{t}^{\ast }$ and ${m}_{l}^{\ast }$ are in the unit of free electron mass (m_0_). For the h-phase AlGaInN, ${m}_{t}^{\ast }$ and ${m}_{l}^{\ast }\,\,$increase uniformly with the increasing mole fractions of Al and Ga. By decomposing the density of states of h-phase quaternary alloys with respect to electron orbitals, dominant contributions from *s*-orbitals are observed at the conduction band minimum. Since the *s*-orbitals have spherical symmetry, the difference between ${m}_{t}^{\ast }$ and ${m}_{l}^{\ast }$ is insignificant even though the atomic configurations of h-phase AlGaInN lack centrosymmetry. Tabulated in Table 3, a similar form of Vegard’s law is exploited to express the electron effective mass numerically in the unit of m_0_ with a bowing parameter of −0.4684 m_0_ and an R^2^ value of 0.990. The ${m}_{t}^{\ast }$ and ${m}_{l}^{\ast }$ of c-phase AlGaInN, except for the Al-rich quaternary alloys, are identical due to the centrosymmetry of zincblende structure. However, both the ${m}_{t}^{\ast }$ and ${m}_{l}^{\ast }$ lift sharply at the crossover points of the direct-to-indirect bandgap transition because the conduction minimum shifts from the Γ-valley to the X-valley at high Al mole fraction, where X-valley has a smaller curvature. At X-valley, the ${m}_{l}^{\ast }$ is significantly greater than the ${m}_{t}^{\ast }$. The drastic difference between ${m}_{t}^{\ast }$ and ${m}_{l}^{\ast }$ is ascribed to the anisotropic nature of *p*-orbitals that dominantly contribute to X-valley, as confirmed by the partial density of states. The electron effective masses of c- and h-phase alloys at Γ-valley are similar. However, with the increasing Ga mole fraction, the electron effective masses of the c-phase alloys become increasingly smaller than those of the h-phase alloys. As a result, although the electron effective mass of c-phase InN is 68% larger than that of h-phase InN, the electron effective mass of c-phase GaN is 9.3% smaller than that of h-phase GaN, which evidences an additional advantage of c-phase GaN in electronic transport.

## Conclusion

In conclusion, lattice constants, bandgaps, and electron effective masses of binary, ternary, and quaternary III-nitrides have been investigated using the accurate and unified LDA-1/2 approach. C-phase III-nitride alloys have smaller bandgaps and electron effective masses than h-phase nitrides. The lattice constants and bandgaps of h- (and c-) phase AlGaInN are shown to follow Vegard’s law with the bowing parameters of −0.082 (0.184) Å and 1.236 (2.406) eV, where the corresponding R^2^ values are 0.998 (0.998) and 0.968 (0.989), respectively. Direct-indirect bandgap crossing points in c-phase AlGaN and AlInN are identified at Al mole fractions of 0.700 and 0.922, respectively. Both the ${m}_{t}^{\ast }$ and ${m}_{l}^{\ast }$ of h-phase AlGaInN are expressed numerically with the bowing parameter of −0.4684 m_0_, where the R^2^ value is 0.990. The electron effective masses of c-phase AlGaInN is found to resemble its h-phase counterparts, except for the Al-rich region because the conduction band minimum is shifted from Γ-valley to X-valley, where X-valley has a heavier electron effective mass. C-phase III-nitrides benefit polarization-free nature making it promising materials for green, blue, and UV emitters. Specifically, h- (and c-) phase In_0.322_Ga_0.678_N (In_0.274_Ga_0.726_N), In_0.162_Ga_0.838_N (In_0.133_Ga_0.867_N), and Al_0.837_In_0.163_N (Al_0.855_In_0.145_N) alloys having the least lattice-mismatch with GaN substrates are shown to be promising active layer materials for green, blue, and UV emitters, respectively.

## Data Availability

The datasets generated during and/or analyzed during the current study are available from the corresponding author on reasonable request.
